# Discovery pipelines for marine resources: an ocean of opportunity for biotechnology?

**DOI:** 10.1007/s11274-019-2685-y

**Published:** 2019-07-02

**Authors:** D. Smith, A. G. Buddie, R. J. M. Goss, J. Overmann, C. Lepleux, M. Brönstrup, B. Kloareg, T. Meiners, P. Brennecke, A. Ianora, F.-Y. Bouget, P. Gribbon, M. Pina

**Affiliations:** 1 CAB International (CABI), Bakeham Lane, Egham, TW20 9TY Surrey UK; 2 School of Chemistry, Biomedical Sciences Research Complex, University of St Andrews, Fife, KY169ST UK; 3 Leibniz-Institut DSMZ-Deutsche Sammlung Von Mikroorganismen Und Zellkulturen GmbH, Inhoffenstraße 7 B, 38124 Braunschweig, Germany; 4 Department of Chemical Biology, Helmholtz Centre for Infection Research, Inhoffenstrasse 7, 38124 Braunschweig, Germany; 5 Station Biologique, CS 90074, 29688 Roscoff CEDEX, France; 6 Leibniz-Institute for Molecular Pharmacology, Robert-Roessle-Strasse 10, 13125 Berlin, Germany; 7 Stazione Zoologica “A. Dohrn” Villa Comunale, 80121 Naples, Italy; 8 Laboratoire D’Océanographie Microbienne (LOMIC) - UMR 7621 CNRS-UPMC, Avenue Fontaulé, 66650 Banyuls sur mer, France; 9 Fraunhofer IME, ScreeningPort, Schnackenburgallee 114, 22525 Hamburg, Germany; 10 CRBIP, Institut Pasteur, 25-28 rue du Dr. Roux, 75015 Paris, France

**Keywords:** Marine, Microorganism, Discovery pipeline, microbial domain Biological Resource Centres (mBRC)

## Abstract

Marine microbial diversity offers enormous potential for discovery of compounds of crucial importance in healthcare, food security and bioindustry. However, access to it has been hampered by the difficulty of accessing and growing the organisms for study. The discovery and exploitation of marine bioproducts for research and commercial development requires state-of-the-art technologies and innovative approaches. Technologies and approaches are advancing rapidly and keeping pace is expensive and time consuming. There is a pressing need for clear guidance that will allow researchers to operate in a way that enables the optimal return on their efforts whilst being fully compliant with the current regulatory framework. One major initiative launched to achieve this, has been the advent of European Research Infrastructures. Research Infrastructures (RI) and associated centres of excellence currently build harmonized multidisciplinary workflows that support academic and private sector users. The European Marine Biological Research Infrastructure Cluster (EMBRIC) has brought together six such RIs in a European project to promote the blue bio-economy. The overarching objective is to develop coherent chains of high-quality services for access to biological, analytical and data resources providing improvements in the throughput and efficiency of workflows for discovery of novel marine products. In order to test the efficiency of this prototype pipeline for discovery, 248 rarely-grown organisms were isolated and analysed, some extracts demonstrated interesting biochemical properties and are currently undergoing further analysis. EMBRIC has established an overarching and operational structure to facilitate the integration of the multidisciplinary value chains of services to access such resources whilst enabling critical mass to focus on problem resolution.

## Introduction

The marine environment provides a huge and, as yet, under-tapped resource for the biotechnology industry (Blasiak et al. 2018). However, whilst individual enzymes/products or their host organisms have been exploited for commercial products, to date there has not been a concerted effort to make use of marine organisms in a more holistic way. Indeed, a recent study highlighted the unevenness of commercial activities in this sector, as may be seen from patents derived from marine organisms: of almost 13,000 sequences of marine origin assigned to patents at the time of writing, 47% were filed by a single multinational company (see Blasiak et al. 2018). Marine science now has a wealth of tools to overcome key bottlenecks in access to and releasing the potential of marine organisms. It has been estimated that less than 1% of novel marine genetic resources will make it to the market place (Royal Society 2017). The massive growth in opportunities afforded by new high throughput technologies enables understanding of the biological and chemical make-up of novel compounds and screening for them in the first place. This has been aided by a similar expansion in computing power and analytical tools in order to put such discoveries into context.

The European Marine Biological Research Infrastructure Cluster (EMBRIC–https://www.embric.eu/) brought six European Research Infrastructures together (Brennecke et al. 2018) and aimed to fulfil one of the key rules for ‘pragmatic blue growth’ described in a recent policy paper by being a ‘well-designed institution’ (Burgess et al. 2018) although marine biotechnology, per se, was notable by its absence in a recent study of the management of ocean resources (Klinger et al. 2018). The European Strategy Forum for Research Infrastructures (ESFRI–https://ec.europa.eu/research/infrastructures/index_en.cfm?pg=home) have encouraged the establishment of Research infrastructures (RIs): facilities, resources and services used by the science community to conduct research and foster innovation.

## Materials and methods overview

There are numerous routes that may be taken in the discovery process. The first step is to select where to sample, followed by the prioritisation of organisms and selective sampling to enrich for organisms with the desired properties. Obviously, in order to invest time and effort wisely, these choices are critical, because the oceans are vast and their potential enormous (Royal Society 2017), but the organisms are difficult to isolate and when they are, they are generally slow-growing. A typical workflow begins with the source of organism, where there are two options:(1) isolation directly from the environment; or (2) selection from those already isolated; the latter for example, may be taken from *ex situ* biological resource collections such as the microbial domain Biological Resource Centres (mBRC—OECD 2007). A number of such mBRCs are coordinated either by the Microbial Resource Research Infrastructure (MIRRI; www.mirri.org/) or the European Marine Biological Research Centre (EMBRC-ERIC) (https://www.embrc.eu/). MIRRI brings together 31 international mBRCs which, together, hold a total of > 330 K microorganisms, ranging from bacteria (including cyanobacteria) to yeasts, filamentous fungi and algae. EMBRC brings together marine biological stations and institutes organised through nine national nodes.

At each level of the discovery process there are technical issues to be addressed. EMBRIC has begun the process of mapping current mechanisms available to help facilitate how the potential of novel organisms is identified and the organisms with the most potential targeted (Brennecke et al. 2018). Options to be considered are for example using metagenomics to assess sample content; using proteomics and metabolomics to identify chemical targets; analysing information on chemical interactions (e.g. allelopathic, grazer defence metabolites) to identify bioactive compounds; and using metabarcoding technology to characterise the species compositions of mass samples of environmental DNA. Bottlenecks could also revolve around compliance with regulation for example obtaining permissions to collect (Kamau et al. 2010; Overmann and Scholz 2017; Smith et al. 2017). Despite numerous attempts to explore the microbial diversity of the planet, to date the majority has yet to be isolated and cultured and consequently has remained largely untapped, especially with regard to bioprospecting (Overmann and Smith 2017). Up to 95% of marine life (by weight) has been estimated to be microbial in nature (Vierros et al. 2016). The failure to recover phylogenetically novel microbial lineages of bacteria and fungi can be attributed to current isolation methodology that is often found to be inadequate (Overmann 2012). Phenotypically novel types of bacteria often have unknown growth requirements and, are highly fastidious, for example: *Myxobacteria*, *Acidobacteria* and *Dehalococcoides ethenogenes* (Foesel et al. 2013; Maymó-Gatell et al. 1997; Sanford et al. 2002). There is a need to employ improved methodology and undertake a systematic approach to culturing microorganisms such as harnessing “culturomics” (Lagier et al. 2012). Dedicated isolation protocols are required, e.g. selective media: to date, less than ca. 1% of all microorganisms can be cultivated (Pan Ming Huang et al. 2012; Overmann et al. 2017). Development of novel approaches to eliciting enhanced production—media development, use of small molecule elicitors, targeted heterologous expression and promoter refactoring. Growth/fermentation technologies need to be expanded, allowing bulking up for production (e.g. Hewitt and Nienow 2010). Once the organism is grown access to the most appropriate methodologies and technologies is needed:

- Immunological ID, Next Generation Sequencing (whole sample; e.g. Chun and Rainey 2014);
- Sequencing; genome, proteins; metabolomics (e.g. Naccache et al. 2014);
- Population/environmental metagenomics (e.g. Lorenz and Eck 2005);
- Bioinformatic tools, data interoperability; Common tools and software (e.g. Okonechnikov et al. 2012);
- Identification of bioactive compounds (e.g. Kim and Mendis 2006).
  In identifying and isolating promising metabolites loss of resources and time by rediscovery of already known compounds must be avoided by using optimal de-replication protocols (e.g. Dias et al. 2012). Supporting technologies are:
- Genome mining of biosynthetic potential (e.g. Wagner-Döbler et al. 2002);
- Bioassay technologies (e.g. Delamarche et al. 2005);
- Routes from crude extract assay hits to scaling, identifying, purifying and structural characterising bioactive compounds (e.g. Li and Vederas 2009) utilising, where possible, genome guided characterisation in complement with spectroscopic guided characterisation.

**Fig. 1:**
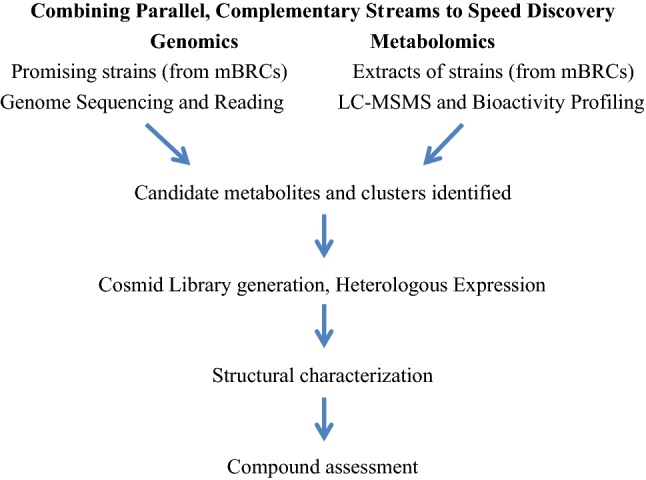
Combining parallel, complementary streams to speed discovery from marine resources

Improved routes from novel bioactive organisms and bioactive extracts, identifying and purifying key bioactives as either a single compound or a synergistic mixture of compounds is needed. In response to this issue EMBRIC is facilitating the search by exploring ways to integrate and mine metabolomic/genomic/metagenomic data. There is also a critical need to bridge the gap between ‘feature identification’ and compound chemical characterisation. Data analysis tools to integrate genome reading with metabolite information are seen as a crucial success factor. Thus, an essential first step is combining parallel, complementary streams to speed discovery (see Fig. 1).

An additional problem is that the majority of microorganisms in the environment are yet to be cultured and this is compounded further by the fact that those from marine environments are renowned slow growers (Joint et al. 2010). In order to address this, DSMZ researchers have been developing culturing technologies for less tractable microbes. Methods under consideration currently include applications already demonstrated in the field e.g. iChip technologies (Nichols et al. 2010) and the single-dilution MicroDrop^®^ technique (Bruns et al. 2003). Nichols et al. (2010) designed an isolation chip (iChip) composed of several hundred, miniature diffusion chambers—each inoculated with a single environmental cell. They showed that microbial recovery in the iChip exceeds many-fold that afforded by standard cultivation, and the species thus obtained are of significant phylogenetic novelty. The method allows access to a large and diverse array of previously inaccessible microorganisms and is well suited for both fundamental and applied research. (e.g. Sherpa et al. 2015). As such, the iChip is a high-throughput platform for parallel cultivation and isolation of previously uncultivated microbial species from a variety of environments. Using a microdispenser system, Bruns et al. (2003) developed a high-throughput innovative technique for the isolation of bacteria in liquid. The MicroDrop^®^ microdispenser system enables the dispensing of highly reproducible volumes (0.5 µl to 2.5 ml) in microtiter plates in less than 1 min per plate allowing the generation of large numbers of bacterial enrichments for downstream cultivation and analysis. Using this technique Gich et al. (2005) obtained 570 bacterial cultures from which the majority was closely related to previously uncultured bacteria. It is necessary to bridge the gap between compound identification and compound chemical characterisation and, ultimately, to gain access to the most appropriate methodologies and technologies. Current “state of the art” approaches utilise High Resolution Mass Spectrometry (MS) and database comparison, coupled with genome analysis. Cloning and heterologous expression has potential but needs considerable further work. Bouslimani et al. (2014) described technologies in mass spectrometry of natural products. A key strength being that very small quantities of sample are required allowing much structural information to be gained from a single mass spectrum. This approach is particularly relevant with the slow-growing marine organisms and shows why such methods are being used to generate information on the selected test strains in EMBRIC’s component laboratories currently. Mass spectrometry structural analysis workflows are emerging that allows faster de-replication and structural elucidation of metabolites, facilitating the screening of new biologically active compounds.

## EMBRIC microbial product discovery pipeline output

To date, the work undertaken has involved the strategic isolation of biotechnologically useful microbes using a DSMZ biofilm method (Gich et al. 2012) and selection of the most promising microbes for extract analysis. DSMZ collected samples of sea water, sediment and sponges from the Pacific Ocean and from over 900 samples isolated, found 248 species considered rare and difficult to grow. These included strains from the phyla Actinobacteria, Bacteroidetes, Proteobacteria and Rhodothermaeota. Two new species of *Arcobacter* have been found and there may be more new taxa to be reported, as characterisation is ongoing. The partners at the University of St Andrews have cloned a large number of gene clusters and used promoter refactoring in attempts to awaken “sleeping clusters”. As a result, they detected novel metabolites demonstrated through the Global Natural Products Social Molecular Networking (GNPS) web-platform.

**Fig. 2:**
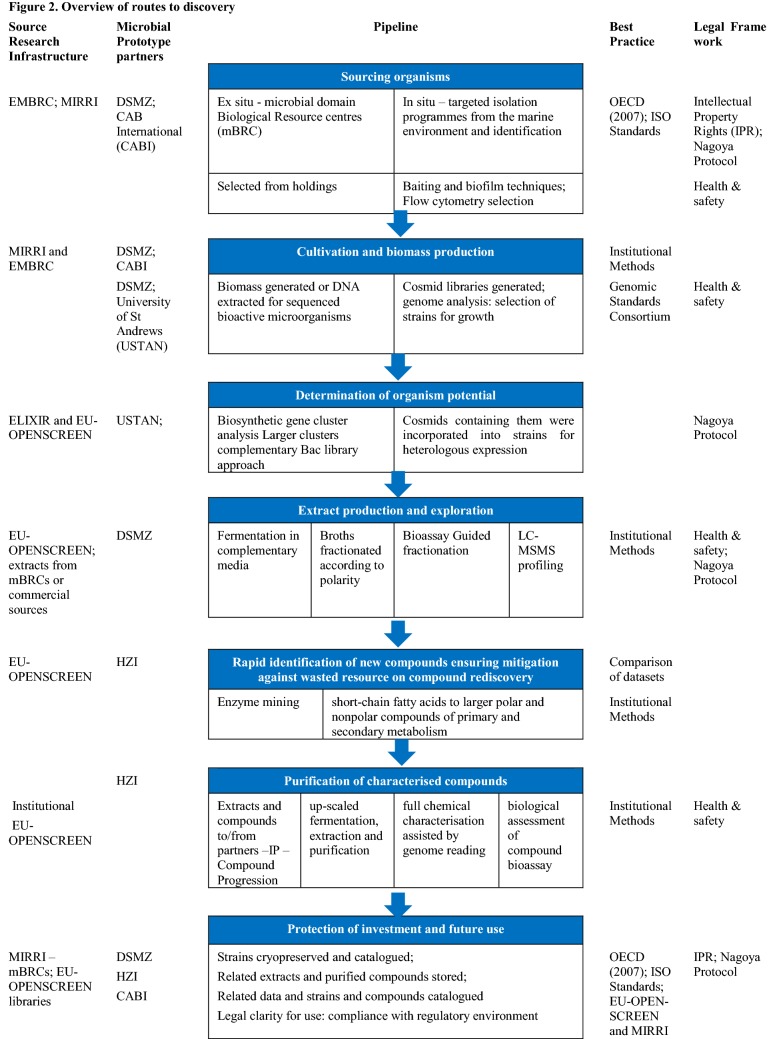
Overview of routes to discovery

Helmholtz-Zentrum für Infektionsforschung (HZI) have received 156 extracts from DSMZ in total comprising pellets, supernatant and resin extracts of 25 strains which have been analysed. The analysis of the LC–MS/MS measurements revealed a total of 557 features detected in at least one of four strains; the number of features was reduced to 312 after discarding features that were found also in the control sample (KM14 growth medium). In-house library matching identified 13 of the 312 metabolites found. Tyramine and Adenine are the metabolites found in each strain with the highest intensity; Glycocholate is present in three strains with very high intensity; 2-Deoxyadenosine is found with the highest intensity in one strain being present only in traces in the other strains (very low intensity); see deliverables on EMBRIC website https://www.embric.eu/deliverables specifically: D6.1 EMBRIC showcases: prototype pipelines from the microorganism to product discovery (Revised 2019) at https://www.embric.eu/sites/default/files/deliverables/EMBRIC%20Deliverable%20D6.1b.pdf. Further analysis of the more recently received strains is ongoing. An overview of the routes to discovery within the EMBRIC project are shown in Fig. 2. Extensive analysis of over 150 cell pellet extracts selected from the 248 strains was carried out via a combination of MS/MS in-house library matching and manual dereplication (using commercial and online opensource databases) allowed the team to assign between 10 and 25% of the detected features to known metabolites.

There were a number of outputs of significance from the microbial prototype pipeline:

- Flow cytometry and biofilm technologies were used for selection of marine organisms in samples that had greatest potential as candidates for biomass production;
- Combined cosmid and BAC library heterologous expression approach developed;
- Genome informed use of isotopes to track new metabolites;
- USTAN generated cosmid libraries for a series of more tractable and sequenced test strains. Biosynthetic gene cluster analysis prioritized nine clusters according to novelty. Cosmids containing them were taken into strains of *Streptomyces coelicolor* and *Streptomyces lividans* for heterologous expression. Fermentation broth extracts revealed heterologously produced metabolites;
- Larger biosynthetic gene clusters were accessed via a complementary BAC library approach, removing the need for cluster stitching prior to heterologous expression;
- Original bottlenecks in getting sufficient DNA for analysis were overcome:
  - Expression level: addressed by introduction of promoters, utilization of different heterologous hosts, and utilization of small molecule elicitors;
  - Metabolite de-replication and assignment: addressed with cluster analysis of LC–MS/MS data aiding the identification of series of closely related compounds;
  - Cyclic peptides difficult to characterize: new approaches explored by pre-incubation with an enzyme;
- HZI have optimized analysis for different metabolite classes: e.g. short-chain fatty acids to larger polar and nonpolar compounds of primary and secondary metabolism. Lessons learned from the metabolome analysis of microalgae in EMBRIC work package 7 (https://www.embric.eu/WP) accelerated their application to the bacteria;
- Use of metabolite clustering analysis with a tool made available as an R package to assist in the identification of novel suites of compounds;
- Use of molecular networking tool from GNPS combined with manual dereplication and matching with in-house library allowed the faster and more efficient annotation of known metabolites. The visualization of the chemical space at the molecular level gave insights on structural relationship between chemical entities in the whole data set, pointing towards potential novel compounds (unknown metabolites);

Raw data and detailed results are to be found on the EMBRIC web site under work package 6 deliverables (https://www.embric.eu/deliverables).

## Discussion

The coordinated activities of the EMBRIC project has demonstrated that workflows across different ESFRI research infrastructures can improve our ability to isolate rare organisms and access their metabolites. The EMBRIC project examined the completeness of the necessary workflows for key types of marine bioproducts and identified some crucial bottlenecks that currently present obstacles to the exploitation of this vast resource. Where possible, strategies to overcome the gaps and problems identified in ways that may benefit the user community have been determined.

When seeking active molecules for use as an effective product, the first step is to identify where the potential solution might be. Are there already organisms, derivatives or even purified compounds that are available (i.e. de-replication) and how might more be discovered in the marine environment? This first step, therefore, may be considered to be *identifying the potential*. Twenty collections registered with the World Data Centre for Microorganisms (WDCM) hold microorganisms sourced from the marine environment; amongst them, they house over 334,000 strains. Even within the EMBRIC partners, there are collections that are not listed on the WDCM; additionally, there are many organisms held by scientists in non-registered collections around the world that are not visible to the researcher. The rest of the 700 + collections in the WDCM hold over two million strains of microorganism, many of which may well originate from the marine environment but there is no single place where all catalogues can be searched and these strains easily revealed. At present, each single collection catalogue would need to be searched and only 85 collections (holding a total of 356,889 strains) are included in the Global Catalogue of Microorganisms (GCM), with only minimal data made available (https://gcm.wfcc.info/). A concerted effort, therefore, is needed to make these strains more readily accessible: a task that can be undertaken by the MIRRI and EMBRIC consortia.

When the species coverage is examined it is very easy to see that there are large gaps between what is known and what is available. There are also estimates that less than 1% of the microorganisms that are in the environment have been discovered, to date. Thus, the question remains: how can this vast resource be tapped? Targeting the organisms and establishing sampling regimes for the chemistry that is needed, is not straightforward. New approaches such as “culturomics” are being harnessed; this entails the exhaustive application of culture media and growth conditions for the maximum recovery of cultivable microorganisms from a biological sample (Lagier et al. 2012). It may also be possible to add to the traditional methods of “baiting” environments with substrates to select specific potential based on chemistry or utilising molecular tools or probes and cloning genes. The danger of simply employing normal isolation techniques to extract and identify the organisms and to get them into axenic mono-culture is that the faster growing and/or more common organisms will be isolated repeatedly whilst completely missing the slow-growing or yet-to-be-cultured ones.

Genomic DNA analysis using next generation sequencing approaches to examine total sample DNA for the yet uncultured is notable way forward but accessing the relevant expertise and facilities for this is not always obvious. Characterisation technologies, data analysis and applications are available and have been identified through this process. The key function of the research infrastructures is to give access to the resources needed for a researcher to make discoveries: e.g. the EMBRIC web tool. ELIXIR, a distributed infrastructure for life-science information: https://www.elixir-europe.org/tools tools offer different ways to find and analyse data to enable such compounds to be identified. The remaining elements in discovery pipelines, scaling-up, extraction, purification and delivery of chemically defined compounds are also elements that have been designed for some specific cases. EU-OPENSCREEN (https://www.eu-openscreen.eu/) in particular has pulled together much of the best practice for chemical screening, and offers long-standing expertise regarding the setup and implementation of chemical screens and subsequent hit-to-lead optimization. EU-OPENSCREEN works closely with collaborating chemists and biologists and provides support throughout the entire screening workflow–from the initial idea to the optimized hit. In the context of EMBRIC, newly isolated marine compounds can be tested against a variety of biological assays using EU-OPENSCREEN in order to obtain bioactivity profiles of newly isolated compounds (e.g., antimicrobial or antiproliferative activities). Moreover, compounds will be transferred into the EU-OPENSCREEN comprehensive compound library, thus increasing European visibility. All of these aspects are organised in such a way that no loss of intellectual property occurs.

A fundamental difficulty in assisting bioindustry to access the most appropriate targets is getting specific information from companies to enable delivery of their needs. To date, resource centres and service providers have tried to second-guess what a company needs, or wants, as many companies are reluctant to let others know what they are working on. Enabling companies to access and utilise information in open databases anonymously misses potential and lessens the opportunity to get the collections and their networks to make more targets available. Appropriate business models with non-disclosure agreements are needed and a common agreement enabled for infrastructure partners to allow others to act on their behalf.

By pooling expertise, a critical mass can be focused on specific user problems, such as, the development of dedicated isolation protocols (e.g. selective media to access the microorganisms that cannot yet be cultivated). In terms of the bottlenecks associated with specific active molecules, gaps between the identification and chemical characterization of compounds can be addressed and appropriate biological assay technology to comprehensively test for bioactivities is available already. Routes from crude extract assay hits to identifying and purifying bioactive compounds are facilitated across the partner research infrastructures. This may be done through access to the most appropriate methodologies and technologies: e.g. common tools and software. By accessing the research infrastructures, the loss of resources and time, by rediscovery of known and already patented compounds, can be avoided (i.e. de-replication). Additionally, this approach ensures compliance with regulations, ensuring that permissions to collect are secured where needed and legal obligations are met. Experts in technology transfer offices across the research infrastructures can also help with expertise or simple information on routes to market, licensing, product marketing and even production in some cases. Above all, research infrastructures must ensure that, in creating such platforms and gateways to technologies and collaborations, they strive for high quality. Information should be collated and incorporated into a web-based tool that helps users negotiate the pipeline through the RI facilities in a manner tailored to user needs. In other words, they must provide the operational tools that meet community best practice with appropriate quality checks and accreditation.

The work of the European Research Infrastructure clusters will continue to evolve through reaction to user needs in order to serve the European Research Area better. Researchers are encouraged to use the resources, expertise and facilities available from the European Research Infrastructures (https://www.esfri.eu/roadmap-2016). In closing, therefore, we note the scale of the task facing stakeholders in the blue bio-economy and commend the multi-partner, international approach encompassed in EMBRIC, as a potential toolkit for enabling the use of the largely untapped marine microbial ecosystem as a source of novel bioactives in a sustainable and responsible manner.
